# Resolvin D5 Inhibits Neuropathic and Inflammatory Pain in Male But Not Female Mice: Distinct Actions of D-Series Resolvins in Chemotherapy-Induced Peripheral Neuropathy

**DOI:** 10.3389/fphar.2019.00745

**Published:** 2019-07-05

**Authors:** Xin Luo, Yun Gu, Xueshu Tao, Charles Nicholas Serhan, Ru-Rong Ji

**Affiliations:** ^1^Center for Translational Pain Medicine, Department of Anesthesiology, Duke University Medical Center, Durham, NC, United States; ^2^Center for Experimental Therapeutics and Reperfusion Injury, Department of Anesthesiology, Perioperative and Pain Medicine, Brigham and Women’s Hospital and Harvard Medical School, Boston, MA, United States; ^3^Department of Neurobiology, Duke University Medical Center, Durham, NC, United States; ^4^Department of Cell Biology, Duke University Medical Center, Durham, NC, United States

**Keywords:** chemotherapy-induced peripheral neuropathy (CIPN), docosahexaenoic acid (DHA), intrathecal injection, neuropathic pain, resolvin D-series (RvDs), sex dimorphism, specialized pro-resolving mediators (SPMs)

## Abstract

Earlier studies have demonstrated that essential fatty acid-derived specialized pro-resolving mediators (SPMs) promote the resolution of inflammation and pain. However, the potential analgesic actions of SPMs in chemotherapy-induced peripheral neuropathy (CIPN) are not known. Recent results also showed sex dimorphism in immune cell signaling in neuropathic pain. Here, we evaluated the analgesic actions of D-series resolvins (RvD1, RvD2, RvD3, RvD4, and RvD5) on a CIPN in male and female mice. Paclitaxel (PTX, 2 mg/kg), given on days 0, 2, 4, and 6, produced robust mechanical allodynia in both sexes at 2 weeks. Intrathecal injection of RvD1 and RvD2 (100 ng, i.t.) at 2 weeks reversed PTX-induced mechanical allodynia in both sexes, whereas RvD3 and RvD4 (100 ng, i.t.) had no apparent effects on either sex. Interestingly, RvD5 (100 ng, i.t.) only reduced mechanical allodynia in male mice but not in female mice. Notably, PTX-induced mechanical allodynia was fully developed in *Trpv1* or *Trpa1* knockout mice, showing no sex differences. Also, intrathecal RvD5 reduced mechanical allodynia in male mice lacking *Trpv1* or *Trpa1*, whereas female mice with *Trpv1* or *Trpa1* deficiency had no response to RvD5. Finally, RvD5-induced male-specific analgesia was also confirmed in an inflammatory pain condition. Formalin-induced second phase pain (licking and flinching) was reduced by intrathecal RvD5 in male but not female mice. These findings identified RvD5 as the first SPM that shows sex dimorphism in pain regulation. Moreover, these results suggest that specific resolvins may be used to treat CIPN, a rising health concern in cancer survivors.

## Introduction

Pain is a cardinal feature of inflammation, as inflammation is often associated with pain (Ji et al., 2016). Mechanisms of pain induction by inflammation and inflammatory mediators have been extensively investigated in the past two decades (Hucho and Levine, 2007; Ji et al., 2014). Inflammatory mediators such as lipid mediators (prostaglandins, epoxyeicosatrienoic acids), nerve growth factor, and proinflammatory cytokines and chemokines (IL-1β, TNF, CCL2, CXCL5) sensitize nociceptors (peripheral sensitization) *via* modulations of ion channels such as voltage-gated sodium channels (Nav1.7, Nav1.8, Nav1.9) and TRP channels (TRPA1 and TRPV1) (Gold et al., 1996; Julius and Basbaum, 2001; White et al., 2005; Binshtok et al., 2008; Constantin et al., 2008; Dawes et al., 2011; Sisignano et al., 2012; Ji et al., 2014). However, our understanding on pain resolution is very limited. Mounting evidence indicates that resolution of acute inflammation is an active process involving the production of specialized pro-resolving mediators (SPMs), such as resolvins, protectins/neuroprotectin, and maresins (Serhan et al., 2008; Spite et al., 2009; Hong et al., 2014; Serhan, 2014; Norling et al., 2016). Resolvin D-series (e.g., RvD1, RvD2) and E-series (e.g., RvE1) are derived from omega-3 polyunsaturated fatty acids docosahexaenoic acid (DHA) and eicosapentaenoic acid (EPA), respectively, and exhibit potent anti-inflammation and pro-resolution actions in various animal models of inflammation and infection (Serhan et al., 2002; Arita et al., 2005; Spite et al., 2009; Ji et al., 2011; Chiang et al., 2012; Buckley et al., 2014). For example, during infection, Resolvin D5 biosynthesis and actions in clearance of bacteria appears to be superior to other resolvins (Chiang et al., 2012) and is a major resolvin produced by human M2 macrophages (Werz et al., 2018).

Studies from different laboratories have also demonstrated potent analgesic actions of resolvins (e.g., RvE1, RvD1, and RvD2) in animal models of inflammatory pain (Bang et al., 2010a; Xu et al., 2010; Lima-Garcia et al., 2011; Park et al., 2011b), postoperative pain (Huang et al., 2011; Wang and Strichartz, 2017; Zhang et al., 2018), and nerve trauma-induced neuropathic pain (Xu et al., 2013b). Mechanistically, RvE1, RvD1, and RvD2 suppressed the processing of nociceptive information in the spinal cord pain circuit (Xu et al., 2010; Park et al., 2011b; Meesawatsom et al., 2016). The analgesic potency of resolvins is 1000 times higher than their precursors DHA and EPA (Xu et al., 2010). Resolvins inhibit pain *via* multiple mechanisms, including immune modulation, glial modulation, and neuronal modulation (Ji et al., 2011). Especially, resolvins (RvD1, RvD2, and RvE1) are potent inhibitors of TRP channels, such as TRPA1 and TRPV1, by acting on sensory neurons (Bang et al., 2010b; Xu et al., 2010; Park et al., 2011b). There is also an association of 17-HDHA, a precursor of D-series resolvins, with heat pain and osteoarthritis pain in humans (Valdes et al., 2017). Compared to RvD1 and RvD2, RvD3, RvD4, and RvD5 are newly established members of resolvin family and their complete stereochemical structures and total organic synthesis were recently achieved (Serhan et al., 2002; Dalli et al., 2013; Winkler et al., 2016; Norris et al., 2017; Norris et al., 2018; Winkler et al., 2018). Very recently, we found that RvD1 and RvD5, but not RvD3 and RvD4, reduced postoperative pain after bone fracture in mice (Zhang et al., 2018). Distinct roles of the DHA-derived resolvins in other pain models had not, to date, been tested. Recently, human tears and human skin blisters have shown sex differences in resolvins and SPM produced *in vivo* (English et al., 2017; Rathod et al., 2017).

Chemotherapy-induced peripheral neuropathy (CIPN) is the dose-limiting toxicity for many commonly utilized classes of anti-cancer agents, such as paclitaxel (Flatters and Bennett, 2004; Li et al., 2014). Neuropathic pain after chemotherapy results in dose reductions or discontinuation of cancer therapy (Cavaletti and Marmiroli, 2010; Sisignano et al., 2014). Given a lack of FDA-approved treatment for CIPN, it is urgent to develop safe and novel treatment for neuropathic pain after chemotherapy. In this study, we directly compared the analgesic actions of D-series resolvins (D1-D5) in a mouse model of CIPN. Our findings not only demonstrated distinct analgesic efficacy of these resolvins but also revealed striking sex dimorphism of these SPMs.

## Materials and Methods

### Animals

Adult CD1 mice of both sexes (25–35 g) were purchased from Charles River Laboratories. *Trpv1* knockout mice (B6.129X1-Trpv1^tm1Jul^/J stock# 003770) and *Trpa1* KO (B6;129P-Trpa1^tm1Kykw^/J stock# 006401) mice were purchased from Jackson Laboratory. All animals were maintained at the Duke University Animal Facility. All the animal experiments performed in this project have been approved by the Animal Care Committee of Duke University.

### Chemotherapy-Induced Neuropathic Pain and Formalin-Induced Inflammatory Pain

To induce CIPN in mice, we performed four intraperitoneal injections of paclitaxel (2 mg/kg per injection, Sigma) on days 0, 2, 4 and 6. To produce inflammatory pain, formalin (5%, 20 μl, Sigma) was injected into the plantar surface of a hindpaw.

### Drugs and Administration

RvD1, RvD2, RvD3, RvD4, and RvD5 were from Cayman Chemical. Ten percent ethanol in PBS was taken as vehicle treatment. For intrathecal (i.t.) injection, mice were briefly anesthetized with isoflurane (2%) and a spinal cord puncture was made between the L5 and L6 levels to deliver reagents (10 μl, 10 or 100 ng SPMs dissolved in 10% ethanol) using a 30G needle. The dose selection of resolvins was based on our previous study (Zhang et al., 2018).

### Behavioral Tests

Animals were habituated for 1–2 h in plastic chambers on an elevated metal mesh floor in the testing environment for at least 2 days before testing. For testing mechanical pain threshold, the plantar surface of each hindpaw was stimulated with a series of von Frey fibers with logarithmically incrementing stiffness (0.02–2.56 g, Stoelting), presented perpendicular to the plantar surface. The 50% paw withdrawal threshold was calculated using Dixon’s up-down method (Dixon, 1980). For the formalin test, pain behavior was recorded every 5 min for 45 min following i.pl. formalin and the duration of licking and flinching in the affected paws was timed (Berta et al., 2014).

### Statistics

All data were expressed as the mean ± SEM. The sample size for each experiment is indicated in the figure legends. All data were analyzed by one-way or repeated measures two-way ANOVA, followed by Bonferroni’s *post hoc* test. p < 0.05 was taken as statistically significant.

## Results

### Intrathecal Resolvins Differentially Inhibit Mechanical Allodynia in Male and Female Mice With CIPN

**Figure 1:**
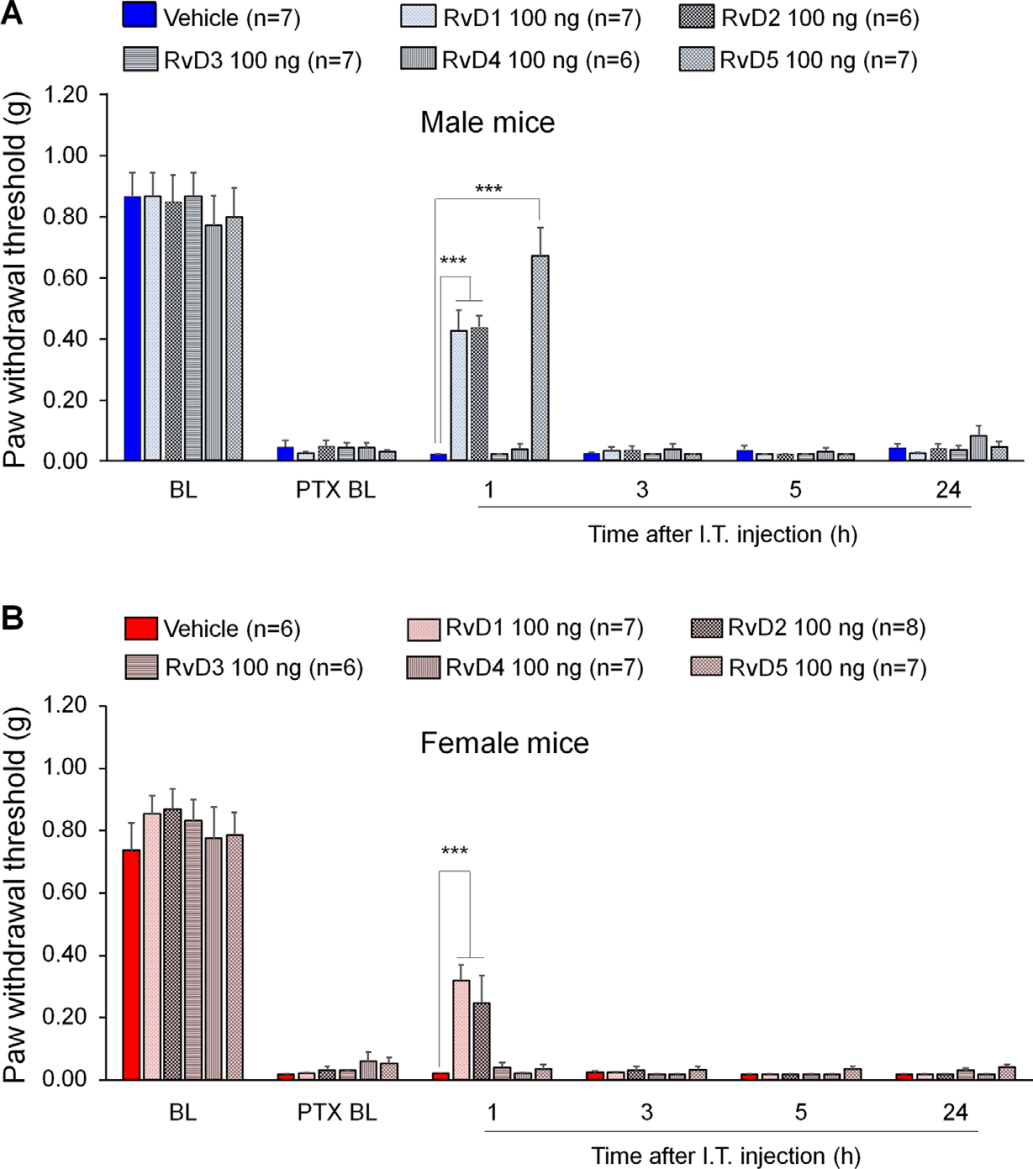
Effects of intrathecal RvD1-D5 on mechanical allodynia in male mice **(A)** and female mice **(B)** 2 weeks after paclitaxel (PTX) injection. Intrathecal RvD1 and RvD2 (100 ng) reduced mechanical allodynia in both sexes. However, intrathecal RvD5 only reduced mechanical allodynia in male mice. ***p < 0.001 versus the vehicle group. Two-Way ANOVA with Bonferroni’s post-hoc test, n = 6-8 mice per sex per group.

We first examined the potential anti-allodynic effects of DHA-derived SPMs, including RvD1, RvD2, RvD3, RvD4, and RvD5 on CIPN compared to vehicle (10% ethanol) *via* intrathecal administration. Intrathecal route is known to target spinal cord cells as well as DRG cells (Yaksh and Rudy, 1976; Ji et al., 2002; Alessandri-Haber et al., 2009). Also, microglial inhibitors and modulators were shown to produce sex-dependent pain inhibition following intrathecal injection (Sorge et al., 2015; Taves et al., 2016). Intrathecal injection of these SPMs (100 ng) was given 2 weeks post the first PTX injection. PTX produced robust mechanical allodynia in both sexes (**Figure 1**). We found that intrathecal resolvins produced different effects on PTX-evoked mechanical allodynia. RvD1 and RvD2 transiently reduced mechanical allodynia in both male and female mice: the effect was observed at 1 h but not 3 h after resolvin injection. Notably, i.t. RvD5 (100 ng) only alleviated mechanical allodynia in male but not female mice. By contrast, RvD3 and RvD4 did not alter mechanical allodynia (**Figure 1A, B**: F_(5, 204)_ = 378.0 in A, F_(5, 210)_ = 438.2 in B, p < 0.001, two-way ANOVA). Together, these results demonstrated distinct analgesic actions of DHA-derived resolvins in CIPN.

### PTX-Evoked Mechanical Allodynia Is Fully Developed in *Trpv1* and *Trpa1* Knockout Mice

**Figure 2:**
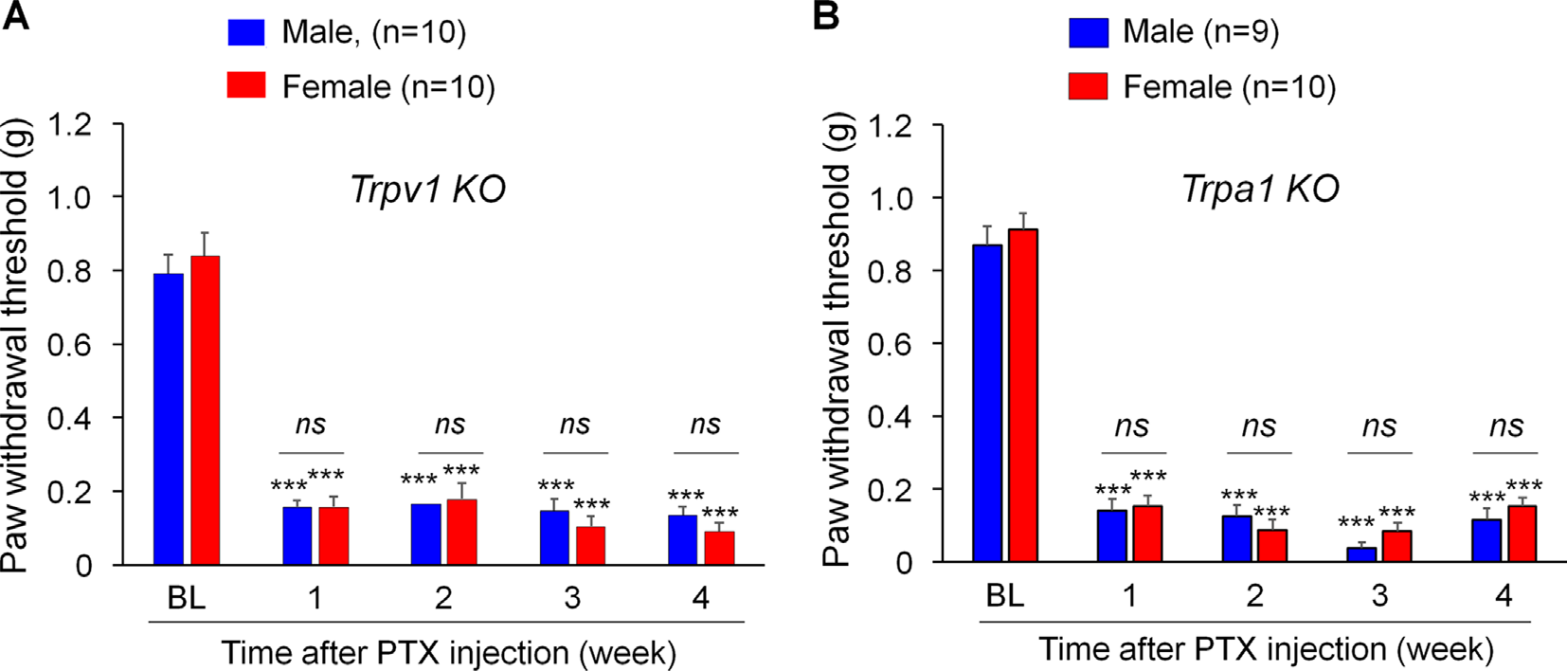
PTX induces sustained mechanical allodynia in *Trpv1* **(A)** and *Trpa1* **(B)** knockout (KO) mice of both sexes. Mechanical allodynia after PTX injection is fully developed in mice lacking *trpv1* (A) and *trpa1* (B) from week 1 and week 4, showing no sex differences. ***p < 0.001 vs. the baseline (BL) control. ns., not significant. Two-way ANOVA with Bonferroni’s *post hoc* test, n = 9-10 mice per sex per group.

TRPV1 and TRPA1 are two critical pain transducers and play important roles in inflammatory pain and neuropathic pain (Caterina and Julius, 2001; Bautista et al., 2006; Constantin et al., 2008; Materazzi et al., 2012). We tested whether TRPV1 and TRPA1 are required for CIPN in a sex-dependent manner using *Trpa1* knockout (KO) mice. Notably, PTX produced remarkable mechanical allodynia in both males and females in *Trpv1* KO mice (**Figure 2A**, F_(4, 90)_ = 140.0, p < 0.001, two-way ANOVA) as well as in *Trpa1* KO (**Figure 2B**, F_(4, 85)_ = 237.6, p < 0.001, two-way ANOVA) mice. Notably, there were no sex differences in PTX-induced allodynia at all the time points we tested (1 to 4 weeks, **Figure 2A and B**).

### Intrathecal RvD5 Inhibits Mechanical Allodynia After CIPN in Male But Not Female Mice Lacking TRPV1 or TRPA1

**Figure 3:**
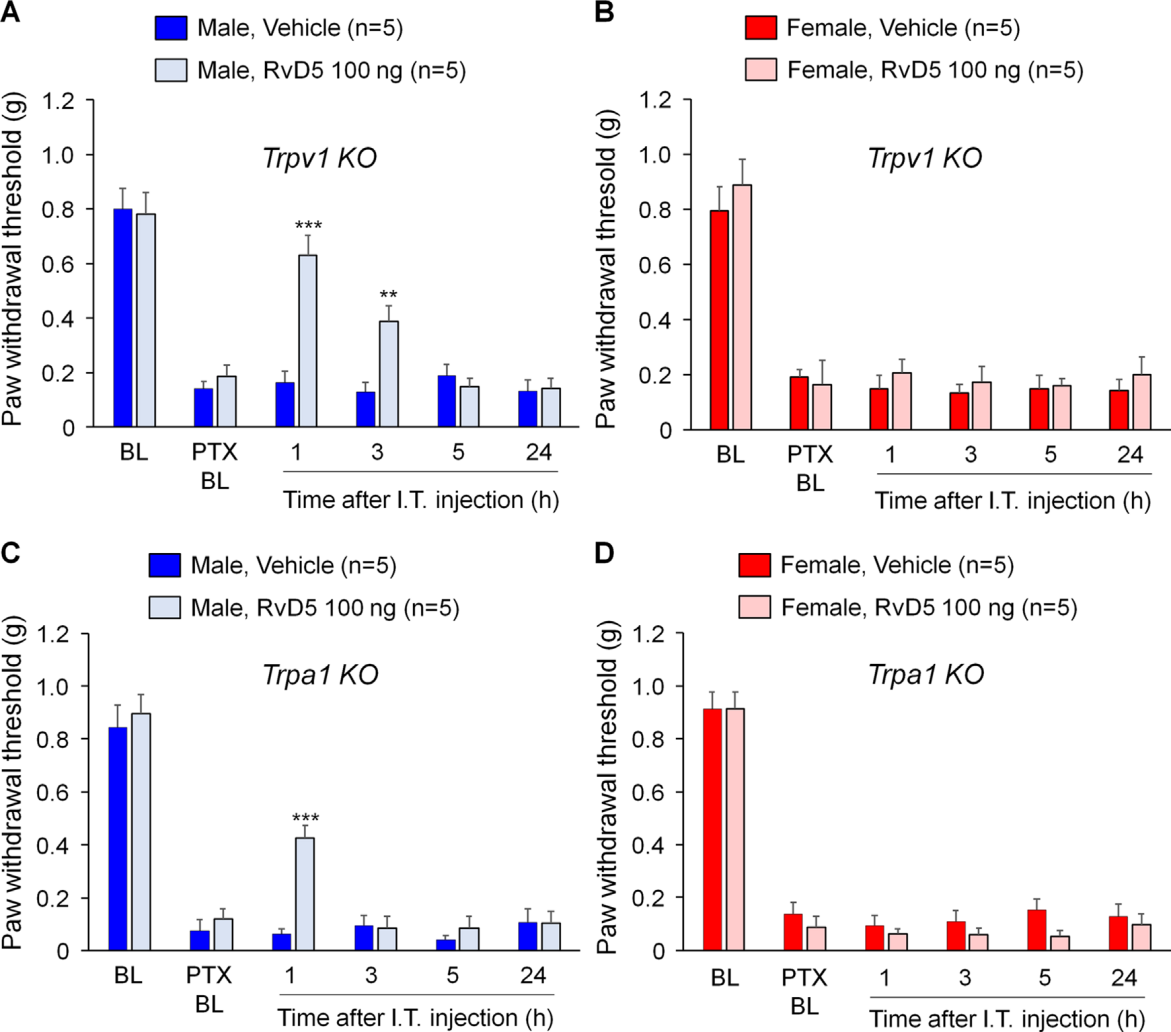
Effects of intrathecal RvD5 on PTX-evoked mechanical allodynia in male mice **(A, C)** and female mice **(B, D)** lacking *Trpv1* **(A, B)** and *Trpa1* **(C, D)**. RvD5 (100 ng, i.t.) was able to inhibit mechanical allodynia in male TRPV1-KO mice **(A)** and male TRPA1-KO mice **(C)**, showing no effects on female mice **(B, D)**. **p < 0.01, ***p < 0.001, vs. vehicle. Two-way ANOVA with Bonferroni’s *post hoc* test, n = 5 mice per sex per group.

Previous studies demonstrated that SPMs are potent inhibitors of TRPV1 and/or TRPA1 (Bang et al., 2010b; Park et al., 2011a; Park et al., 2011b; Serhan et al., 2012). To test the hypothesis that RvD5 reduced PTX-induced neuropathic pain through TRPV1 or TRPA1, we tested the analgesic action of RvD5 in *Trpv1* and *Trpa1* KO mice. We found that i.t. RvD5 (100 ng) reduced mechanical allodynia in male but not female* Trpv1* KO mice in comparison to the vehicle treatment (**Figure 3A, B**: F_(5, 48)_ = 47.54 in A, F_(5, 48)_ = 42.63 in B, p < 0.01, two-way ANOVA). Similarly, i.t. RvD5 inhibited PTX-induced pain in male but not female *Trpa1* KO mice (**Figure 3C, D**: F_(5, 42)_ = 76.65 in C, F_(5, 48)_ = 121.9 in D, p < 0.001, two-way ANOVA). Taken together, these results implicated that anti-allodynic effects of RvD5 against CIPN do not require TRPV1 or TRPA1.

### RvD5 Reduces Formalin-Induced Spontaneous Pain Only in Male Mice

**Figure 4:**
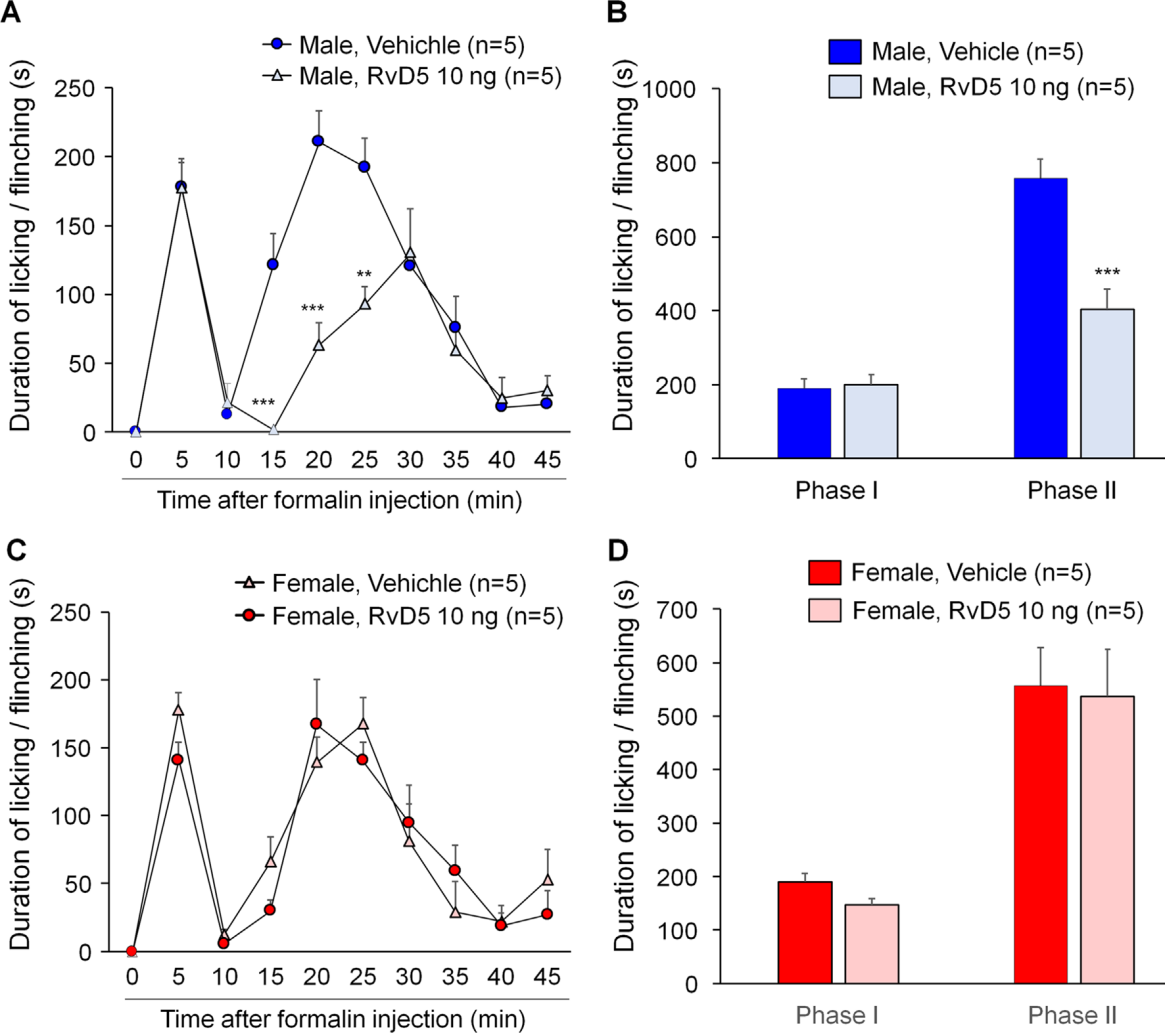
Intrathecal RvD5 reduces formalin-induced inflammatory pain in male mice **(A, B)** but not female mice **(C, D)**. **(A, C)** Time course of formalin-induced pain in males **(A)** and females **(C)** with intrathecal vehicle or RvD5 (10 ng), given 30 min prior to formalin injection. **(B, D)** Phase I (0-10 min) and Phase II (10-45 min) responses in males **(B)** and females **(D)**. Note that RvD5 only inhibits Phase II response in males but has no effect on Phase I response in both sexes. **p < 0.01, ***p < 0.001 versus vehicle group. Two-way ANOVA with Bonferroni’s *post hoc* test, n = 5 mice per sex per group.

To further confirm sex dimorphism of RvD5-produced anti-allodynic effects, we tested an inflammatory pain model induced by intraplantar formalin injection. Male and female mice were pre-treated by i.t. RvD5 (10 ng) prior to formalin treatment (5% in PBS, 20 μl, i.pl). The results of formalin-induced spontaneous pain were presented as phase I (0–10 min) and phase II (10–45 min after i.pl. formalin). Intrathecal RvD5 (100 ng) significantly reduced Phase II behavior in male mice (**Figure 4A, B**: F_(8, 72)_ = 21.34 in A, F_(1, 16)_ = 71.39 in B, p < 0.01, two-way ANOVA), without effects on Phase I behavior. However, i.t. RvD5 failed to affect neither Phase I nor Phase II behavior in females (**Figure 4C, D**: F_(8, 72)_ = 18.18 in C, F_(1, 16)_ = 36.44 in D, p > 0.05, two-way ANOVA). These results confirmed a male-dominant analgesic effect of RvD5 in an inflammatory pain.

To determine if RvD3 would produce a rapid and transient analgesia in inflammatory pain, we tested the actions of intrathecal RvD3 (100 ng) in both male and female mice using the formalin test. Our data indicated that intrathecal RvD3 (100 ng) did not affect Phase I or Phase II behavior in male mice (vehicle group: 135.2 ± 3.83 s in Phase I and 528.8 ± 71.38 s in Phase II; RvD3 group: 208.2 ± 21.57 s in Phase I and 653.0 ± 122.86 s in Phase II, F (1, 16) = 42.51, p > 0.05, two-way ANOVA, n = 5 mice per group). Neither did RvD3 produce any analgesic effects in female mice (vehicle group: 265.0 ± 39.37 s in Phase I and 962.8 ± 110.63 s in Phase II; RvD3 group: 307.4 ± 52.83 s in Phase I and 973.8 ± 129.47 s in Phase II, F (1, 16) = 69.77, p > 0.05, two-way ANOVA, n = 5 mice). Together, our results suggest that RvD3 has no apparent analgesic actions in inflammatory pain, neuropathic pain (**Figure 1**), and postoperative pain (Zhang et al., 2018).

## Discussion

SPMs have been shown to produce pain relief in inflammatory pain (Svensson et al., 2007; Bang et al., 2010a; Xu et al., 2010; Lima-Garcia et al., 2011; Park et al., 2011b), postoperative pain (Huang et al., 2011; Wang and Strichartz, 2017; Zhang et al., 2018), and nerve trauma-induced neuropathic pain (Xu et al., 2013a; Xu et al., 2013b). CIPN is a unique type of neuropathic pain, as there is very limited activation of microglia in the spinal cord after CIPN by PTX (Zheng et al., 2011; Robinson et al., 2014). In contrast, nerve injury/nerve trauma produces remarkable microglial activation and microgliosis in the spinal cord (Echeverry et al., 2008; Suter et al., 2009; Clark et al., 2012). Furthermore, spinal blockade of p38 MAP kinase reduced nerve trauma-induced mechanical allodynia but failed to reduce PTX-induced mechanical allodynia (Taves et al., 2016; Luo et al., 2018). In this study, we tested the analgesic actions of DHA-derived SPMs (resolvin D1-D5) in a mouse CIPN model induced by paclitaxel and had two interesting findings. First, D-series resolvins showed different analgesic efficacy in male mice: intrathecal injection of RvD1, RvD2, and RvD5 (100 ng) reduced PTX-induced mechanical allodynia, whereas RvD3 and RvD4 treatment had no effect. RvD3 and RvD4 (100 ng, i.t.) also failed to inhibit postoperative pain after bone fracture (Zhang et al., 2018). However, inflammatory pain is much more sensitive to SPMs. For example, 10 ng (i.t.) of RvE1 is sufficient to reduce formalin-induced inflammatory pain (Xu et al., 2010) but 100 ng RvE1 is needed to inhibit neuropathic pain (Xu et al., 2013b). Also, intrathecal RvD5 at a lower dose (10 ng) was able to reduce formalin-induced inflammatory pain (**Figure 4**), as compared to higher dose (100 ng) for treating neuropathic pain. Second, the analgesic action of RvD5 is sex-dependent: RvD5 only reduced mechanical allodynia after CIPN in male mice, showing no pain relief in female mice. This sex dimorphism of RvD5’s analgesia was also demonstrated in another pain model: formalin-induced inflammatory pain was reduced by i.t. RvD5 in male but not female mice. This is the first report of sex dimorphism in SPM-induced analgesia in any pain model.

Despite higher incidence of chronic pain in women, majority of preclinical studies on pain used male animals (Mogil, 2012; Chen et al., 2018). To our knowledge, previous studies on SPM regulation of pain were all conducted in male mice and male rats. Mechanisms underlying sex dimorphism in chronic pain are unclear. Multiple social, psychological, and biological factors have been proposed (Riley et al., 1998; Mogil, 2012; Fillingim et al., 2009). Of note, the neuro-immune interactions are considered an important contributor to sex dimorphism in chronic pain (Sorge et al., 2015). Recent evidence suggests that spinal microglia regulate neuropathic pain and inflammatory pain only in male mice (Sorge et al., 2015). For example, spinal injection of microglial inhibitor minocycline reduced neuropathic pain only in male mice (Sorge et al., 2015; Chen et al., 2018). Blockade of microglial signaling with inhibitors of P2X4, TLR4, and p38 MAP kinase *via* intrathecal injection also inhibited neuropathic pain in male but not female mice (Sorge et al., 2011; Sorge et al., 2015; Taves et al., 2016; Luo et al., 2018). Sex dimorphism in the pathogenesis of CIPN is largely unknown. Given the limited role of spinal microglia in PTX-induced CIPN (Zheng et al., 2011; Robinson et al., 2014; Luo et al., 2018), other immune cell types such as macrophages and T cells should play an important role in CIPN. For example, macrophage infiltration and activation in DRG and sciatic nerve promotes the development of CIPN (Liu et al., 2014; Liu et al., 2016; Montague and Malcangio, 2017; Montague et al., 2018). T cells appear to regulate both the induction and resolution of CIPN (Liu et al., 2014; Krukowski et al., 2016). Although resolvins have been shown to inhibit TRPA1 and TRPV1 in inflammatory pain (Park et al., 2011b), TRPA1 and TRPV1 are not required for the development of CIPN (**Figure 2**). Consistently, mechanical allodynia after CIPN requires large Aβ-fibers but not C-fibers (Xu et al., 2015). Furthermore, the anti-allodynic effect of RvD5 retained in mice lacking TRPA1 and TRPV1 (**Figure 3**). Thus, intrathecal injection of RvD5 may target immune cells in DRGs for producing sex-dependent analgesic actions. Notably, macrophage signaling in DRGs appears to regulate CIPN in a sex-dependent manner (Luo and Ji, In press).

The D-series resolvins produce their beneficial actions *via* specific G protein coupled receptors, such as GPR32 for RvD1 and RvD5, and GPR18 for RvD2 (Krishnamoorthy et al., 2010; Chiang et al., 2012; Chiang et al., 2015), and specific unique receptors for RvD3 and RvD4 remain to be identified. Nonetheless, RvD3 has been found to activate human GPR32 *in vitro* (Dalli et al., 2013). It is surprising that only RvD5 but not RvD1 showed sex-dependent analgesia in CIPN, since both RvD1 and RvD5 share the same receptor (GPR32). It is possible that RvD1 and RvD5 may differently activate GPR32 in resolving pain. Moreover, additional specific receptors for RvD5 might exist in immune cells (e.g., macrophages) that mediate RvD5-induced pain reduction in males. Notably, RvD5 is a major resolvin produced by human M2 macrophages (Werz et al., 2018). Intrathecal RvD5 may also act on M2 macrophages to produce anti-inflammatory cytokines (e.g., IL-10) for pain relief (Bang et al., 2018). Sex dimorphism in SPMs was also revealed in recent human studies. For example, human emotional tears and human skin blisters have shown sex differences in resolvins and SPM produced *in vivo*, as well as the rate of resolution of inflammation (English et al., 2017; Rathod et al., 2017). Hence, it is not unexpected that sex differences are present in the actions of SPMs, which may also reflect sex differences in the expression patterns of their specific receptors. Future studies are warranted to investigate the analgesic actions of RvD5 in GPR32 knockout mice and identify additional receptors of RvD5 in different sexes of mice with CIPN. Future studies are also needed to investigate how sex hormones regulate the production of resolvins and the actions of resolvins.

In conclusion, CIPN is a rising health concern in the world due to increasing number of cancer survivors. There is a lack of approved treatment for CIPN. Our findings suggest that different resolvins may be used to treat CIPN in cancer patients and improve the life quality of millions of cancer survivors in different sexes.

## Data Availability

The raw data supporting the conclusions of this manuscript will be made available by the authors, without undue reservation, to any qualified researcher.

## Ethics Statement

The animal studies were approved by Duke IACUC.

## Author Contributions

XL, YG, and XT did the experiments and analyzed the data. R-RJ, XL, and CS wrote the paper.

## Funding

The work was supported by US National Institutes of Health grants R01DE17794, R01NS87988, and R21NS91779 to R-RJ. CS is supported by National Institutes of Health grant R01GM038765.

## Conflict of Interest Statement

The authors declare that the research was conducted in the absence of any commercial or financial relationships that could be construed as a potential conflict of interest.
